# Caring touch as a bodily anchor for patients after sustaining a motor vehicle accident with minor or no physical injuries - a mixed methods study

**DOI:** 10.1186/s12906-016-1084-2

**Published:** 2016-03-22

**Authors:** Fanny Airosa, Maria Arman, Tobias Sundberg, Gunnar Öhlén, Torkel Falkenberg

**Affiliations:** Karolinska Institutet, Institution of Neurobiology, Care Science and Society, Huddinge, Sweden

**Keywords:** Emergency department, Healing touch, Integrative care, Mixed methods, MVA, Pain, Tactile massage

## Abstract

**Background:**

Patients who sustain a motor vehicle accident may experience long-term distress, even if they are uninjured or only slightly injured. There is a risk of neglecting patients with minor or no physical injuries, which might impact future health problems. The aim of this study was to explore patients’ subjective experiences and perspectives on pain and other factors of importance after an early nursing intervention consisting of “caring touch” (tactile massage and healing touch) for patients subjected to a motor vehicle accident with minor or no physical injuries.

**Methods:**

A mixed method approach was used. The qualitative outcomes were themes derived from individual interviews. The quantitative outcomes were measured by visual analogue scale for pain (VAS, 0-100), sense of coherence (SOC), post-traumatic stress (IES-R) and health status (EQ-5D index and EQ-5D self-rated health). Forty-one patients of in total 124 eligible patients accepted the invitation to participate in the study. Twenty-seven patients completed follow-up after 6 months whereby they had received up to eight treatments with either tactile massage or healing touch.

**Results:**

Patients reported that caring touch may assist in trauma recovery by functioning as a physical “anchor” on the patient’s way of suffering, facilitating the transition of patients from feeling as though their body is “turned off” to becoming “awake”. By caring touch the patients enjoyed a compassionate care and experience moments of pain alleviation. The VAS pain ratings significantly decreased both immediately after the caring touch treatment sessions and over the follow-up period. The median scores for VAS (*p* < 0.001) and IES-R (*p* 0.002) had decreased 6 months after the accident whereas the EQ-5D index had increased (*p* < 0.001). There were no statistically significant differences of the SOC or EQ-5D self-rated health scores over time.

**Conclusions:**

In the care of patients suffering from a MVA with minor or no physical injuries, a caring touch intervention is associated with patients’ report of decreased pain and improved wellbeing up to 6 months after the accident.

**Trial registration:**

ClinicalTrials.gov Id: NCT02610205. Date 25 November 2015.

## Background

Motor vehicle accidents (MVAs) are common and 1.24 million people die each year as a result of road traffic crashes and between 20–50 million more people suffer from non-fatal injuries worldwide [1]. In Sweden, about 12 000 individuals are admitted to hospital annually, due to injuries caused by a MVA. Many of those who seek care at emergency departments are diagnosed with minor or no physical injuries [2]. Mayou and Bryant [3] highlight the risk of neglecting patients with minor or no physical injuries, so as to avoid chronic health problems in the future. In contrast to the common notion that patients with minor or no physical injury do not need active rehabilitation or follow-up, Ottosson et al. [4] found that patients with minor injuries reported a low recovery rate and that those patients more often suffered from anxiety or were depressed at the time of the injury. The most common psychological impacts following MVAs are post-traumatic stress disorder (PTSD), depression, anxiety and chronic pain [5–9]. It seems that females more often than males suffer from mental stress following an MVA [10]. PTSD may develop in people who are exposed to life-threatening events such as violence, natural disasters or other extremely stressful events [11]. Caring for patient’s existential needs may be neglected in today’s emergency departments, especially after the initial caregiving [12]. A previous study indicated that patients admitted to a short-term emergency ward might benefit from a caring touch intervention, e.g., tactile massage or healing touch aiming to decrease stress, pain, anxiety and to create a sense of being secure [13]. However, there are as yet no published studies on the potential benefits of caring touch after an MVA with minor or no physical injuries, and how this might be perceived by patients and affect self-reported pain levels, sense of coherence (SOC), posttraumatic stress disorder (IES-R) and health (EQ-5D) when discharged directly home after examination at the emergency room.

## Methods

### Aim

The aim of this study was to explore patients’ subjective experience and perspectives on pain and other factors of importance after an early nursing intervention consisting of “caring touch” (tactile massage and healing touch) for patients subjected to a motor vehicle accident with minor or no physical injuries.

### Study design and setting

The study was conducted as a single-arm longitudinal observational study, combining qualitative and quantitative perspectives, i.e. a mixed-methods design. The rationale for combining quantitative and qualitative methods was to provide a comprehensive exploration of the research question [14]. The study was conducted at the emergency care department of a large university hospital in Stockholm County, Sweden.

### Participants

A recruitment of potential study patients was made up from a list of incoming patients, inclusion criteria were; 18 years and above, literate in Swedish and cognitively intact, arriving at the emergency department following an MVA, and who upon medical examinations were given an injury severity score (ISS) between 0–3 and subsequently discharged straight home. ISS is a 0–8 point scale rating injury severity, where a rating of 0 indicates no physical injury, 1–3 represents minor physical injuries; and 8 corresponds to a life-threatening injury [15].

Patients were invited to participate based on a convenience sampling procedure. Initially, the sample of participants was estimated in relation to the possibility of having a control group receiving conventional care only. However, due to logistical barriers in the clinical setting it was not possible to include a proper control group. The patients were informed about the study by mail during the week after the MVA, and those interested in participating in the caring touch intervention were asked to contact the author (FA) by phone or mail and subsequently completed a written informed consent form during the first encounter with the therapist.

### Intervention with caring touch

In this study we explore patients experience of a caring touch and not the treatment per se. Touch can be very private and intimate, and for this reason the patients could choose between tactile massage, which requires direct contact with the patient’s skin using a vegetable oil, or healing touch, which can be performed without direct contact with the patient’s skin. In this way, patients who were ambivalent about having their skin touched directly could choose the fully-clothed option. All of the participants choose to receive tactile massage in the beginning of the treatment period and ten percent of the participants later decided to try the treatment with healing touch. The caring touch was provided by nursing staff in a special treatment room with soft lighting and music, with the patient lying on a massage table. The tactile massage was performed by three assistant nurses certified in tactile massage with the same qualifications. Healing touch was given by a nurse certified at level four (of five) in Healing Touch. These two particular touch therapies were selected due to the fact that they were already being provided at the emergency department. The caring touch was adjusted to suit each patient and lasted for 20–60 min (mean = 45 min), once a week, for a maximum of eight treatment sessions altogether. The tactile massage, i.e. a soft tissue massage, was intended to stimulate touch receptors in the superficial layers of the skin and underlying tissues, without applying direct pressure or stretching to the muscles [16]. The massage can be described as slow, gentle, structured, circulating movements with the palm of the therapist’s hand, during which natural oil, or oil with the fragrance of lavender, was applied, sometimes to the whole body. The patient was embedded in towels and blankets, with only the body part undergoing treatment being uncovered.

The healing touch was based on an established procedure, during which the therapist applied a light pressure to the feet, ankles, knees, hips, stomach, heart area, arms, throat, forehead and scalp [17] with the benefit that the patient could be fully dressed during the healing touch, as the nurse used her/his hand in different positions on the patient’s body.

### Data collection and analysis

Data was collected from September 2012 through May 2014 with a hold-up during June to August, and during Christmas, due to the therapists’ vacation. The questionnaires were administrated at inclusion and at follow-up by mail after 6 months. VAS pain ratings were measured before and after each treatment. Individual interviews were conducted after 3 months.

#### Interviews

The first author (FA) conducted the interviews in a closed meeting room at the hospital, 3 months after the patient’s first hospital visit. The time span was set to allow the patients to conclude their treatments with caring touch. The interviews, which lasted up to 60 min, were conducted in Swedish, digitally recorded and transcribed verbatim by the first author. An open-ended question was used to initiate the interviews: “Please tell me what you experienced when you had your motor vehicle accident” with an additional question, “Please tell me what you experienced when you received the caring touch”. During the interviews, there were continuous follow-up questions like “could you please explain further” and “could you give me an example”. During the interview, questions like “do I understand you right when you said…” were asked for clarification.

The interviews were analysed using Systematic Text Condensation (STC), based on Giorgi’s psychological phenomenological analysis [18]. Applying a phenomenological approach, one looks at the objects from the perspective of how they are experienced, while bracketing presuppositions, allowing the essence of the phenomenon to emerge. The procedure consisted of the following steps: 1) An overview of the data was established, reading through all the interview transcripts while trying to bracket preconceptions, the purpose being to get an overall impression – “from chaos to themes”; 2) A systematic review of the interviews was made line by line, identifying and sorting meaning units, in order to elucidate the research question – “from themes to codes”; 3) Meaning units were systematically abstracted and sorted into thematic code groups across individual patients – “from code to meaning”; 4) Data was synthesized from the thematic code groups to descriptions and concepts – “from codes to concepts”. Two authors (FA and MA) independently did a preliminary reading and then discussed the meaning of the text. STC as well as Giorgi’s method implies an analytic reduction with shifts between de-contextualization and re-contextualization of data.

#### VAS pain ratings and questionnaires

The quantitative outcome was current level of pain measured by VAS, ranging from 0 (no pain) to 100 (worst imaginable pain) [19]. Patients rated their current VAS pain at baseline during the initial visit and before and after each treatment session with caring touch, and then again via a postal follow-up after 6 months. The VAS is a standard instrument for assessing pain that was feasible for the nurses to use in the emergency care setting.

Further exploratory outcomes were sense of coherence (SOC), constructed by Antonovsky on the basis of a salutogenic model. The Sense of Coherence scale was of interest since this instrument capture the patients’ experiences of comprehensibility, manageability, and meaningfulness which we assumed could be linked to patient recovery. SOC, a 13-item rating scale, developed using the subscales of comprehensibility, manageability, and meaningfulness. Total scores of 21–59 indicate low sense of coherence, 60–74 an average sense of coherence, and 75–91 high sense of coherence [20]. Additionally, the Impact of Event Scale (IES-R) has been well used in previous trauma research and was decided as being an appropriate tool to explore to what extent the patients experienced post-traumatic stress disease. IES-R, 22-item scale shows the degree to which the traumatic experience is felt on a consciousness level, and if the person exhibits avoidant behaviour. The IES-R is based on a 4-point frequency scales (i.e., 0 = not at all, 1 = a little bit, 2 = moderately, 3 = quite a bit, and 4 = extremely). An average of the total scale sum of 1.8–2.0 indicates post-traumatic stress disorder. The IES-R seems to be a solid measure of post-traumatic phenomena that can augment related assessment approaches in clinical and research contexts [21, 22]. The European Quality of Life (EQ-5D) instrument was employed to explore patients’ health-related quality of life and self-related health. The EQ-5D instrument was selected because of the short-form and that it has been widely used to measure quality of life among the County Councils of Sweden. EQ-5D is a standardized instrument for measuring health outcome. Respondents classify their health in terms of five dimensions: mobility, self-care, usual activities, pain/discomfort, and anxiety/depression. Each dimension has three levels of severity: (1) no problems, (2) moderate problems, and (3) severe problems. From the sum a number of total 243 combinations of health can be created. Each health combination generates an index value from -0.59 to 1.0, where 1.0 indicates full health. Additionally, the EQ-5D has a visual analogue scale for self-rated health with the anchors at zero (worst imaginable health) up to 100 (best imaginable health) [23].

Data from VAS pain ratings and questionnaires was manually transferred from paper into an electronic database before statistical analysis. Summary characteristics of patients were presented as proportions, mean, median, standard deviation and/or min-max values. Change scores of VAS pain ratings, SOC, IES-R and EQ-5D over time between baseline and follow-up after 6 months were analysed for patients with complete data. Considering rating scales and ordinal types of data, and the relatively small sample sizes, non-parametric statistical analysis, i.e. the Wilcoxon signed-rank test was employed for assessing change scores over time. All p-value calculations were conducted with a 5 % significance level. An additional descriptive analysis was conducted for VAS pain ratings before and after each treatment session with caring touch. Computational software included STATA 13, StataCorp, USA and Microsoft Excel 2011, Microsoft, USA.

## Results

**Fig. 1:**
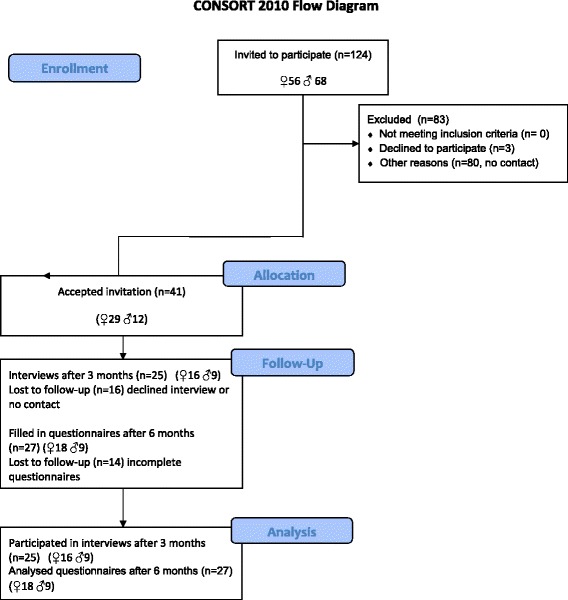
Flowchart of patients in the study.

In total, 124 eligible MVA patients were invited to participate in the study, and 41 patients accepted to participate. The flow of patients through the study is detailed in Fig. 1.

A larger proportion of women accepted to participate (*n* = 29/41; 70 %) in the study. Three months after study inclusion 25 patients volunteered for interviews and 27 patients completed the questionnaires after 6 months. Additional characteristics of the patients are given in Table 1.

**Table 1:** Patient characteristics

Patients, n (%)	41 (100 %)
Women, n (%)	29 (70 %)
Age: mean (sd); median (min-max)	42 (14) years; 41 (19–69) years
Caring touch interventions of; tactile massage healing touch	41 (100 %), 5 (10 %) of those had also tried healing touch

### Interviews

Four themes emerged from the qualitative data; *a way of suffering*, *caring touch as a bodily anchor, enjoying compassionate care and moments of pain alleviation.*

#### A way of suffering

In the beginning of the trauma, some patients experienced their body as “turned off”, and then slowly coming “awake”. This theme includes the first reaction of unreality when the accident happened: *“You know you get a shock, you just stand shivering”* and feelings of surprise and panic, as well as experiencing that time stops and perceptions become clearer and stronger: *“My heart was beating, I was shaking, could not think clearly, I was terrified”*. This could also be expressed as a surrealistic feeling of being in a movie, not really aware of what is going on: *“I was not prepared, everything was so strange. I don’t know… everything got blurred it was like being in a film or something”*. Some experienced instant feelings of anger towards the person responsible for the accident: *“I became very angry at the person who caused the accident!”* and thoughts of fear of being disabled or even dying came up: “*I thought I would die or become a package… it was the most horrifying experience I have ever had”*. Trying to be practical and take control over the situation, patients said: “*Clearly I did not care about myself, my first thought concerned the car, I am unemployed and no car and I need to pick up my son, that’s what I thought about…”.* Later, patients experienced their bodies “waking up”, feeling pain, numbness or stiffness in their body, a body which was changed: *“I had bruises after the belt, and I did not recognize my body, it was difficult to take a shower and look at myself, it felt very uncomfortable”.*

Patients reported a sense of energy loss and a feeling of fear. Weakness appeared and pain started to invade the body.

Taken to hospital, patients felt secure in the trauma room, experienced the staff as professional, trusting and caring: *“When I arrived at the emergency room, I realized that I might be severely injured… I cried and laughed… everyone was so nice, they stayed with me the entire time…they were really nice”.* But when all the examinations were completed, and it became clear that they had not sustained any physical injuries, some of the patients experienced that they were no longer of interest to the staff. They were put in a corridor or a small room without any way of getting the staff’s attention: *“But when the x-ray was finished and it was clear that I was OK, they put me in a small room and nobody cared about me any longer”*. Patients felt that they suddenly became unimportant and they felt forgotten, abandoned and alone, without any way of getting their needs met: *“You were put in a small compartment and you were “nobody”… They pulled down the curtain and then “you”, the patient, the victim disappeared… I felt abandoned”*. Later discharged home in many cases alone with fearful thoughts: *“It was so scary, when I was back home, my God do I dare to move…will I lose my mind…what about my neck!…?”* Many of the patients suffer from pain 3 months after the accident and find it obstructed from being physically active. The pain disturbs their sleep and they feel physically weak and have trouble concentrating. Some patients also felt a lot of frustration and bitterness, that the person who caused the accident had got away too easily. This could be voiced as identifying the other person involved in the accident as responsible: *“I did not cause the car accident. It was the woman’s fault…she shouldn’t have turned, turning was prohibited! …she got away too easily, I took the beating so unfair…”.* Almost all of the patients experienced an intensified awareness of traffic, both physically and mentally. They tended to look more often in their rear-view mirror, expecting someone to collide with them. Some patients got severe, traffic-related problems in terms of their heart rate increasing, as well as feeling stress and anxiety. Most of them drive more carefully today: *“You are very frightened afterwards when you drive, you look carefully at every car…I just sit and look if the car behind me is slowing down or not, I am terrified!”.*

#### Caring touch as a bodily “anchor”

A common experience for all the patients was the disappearance of negative thoughts and pain when receiving caring touch: *“This massage is about the soul, after the accident the soul is damaged, the body is there…it is about your soul and your mind, and massage helps that part, of course helps against pain, but most of all it’s the part touching your soul, you relax, you forget, you become as you once were”.* Caring touch involves dimensions of the body and soul. During a trauma, patients experienced their body and soul as being “scattered”. Patients became aware of this disconnect between body and mind during the caring touch treatments: “*And then the treatment put together all the scattered pieces of my puzzle back”,* and gave them a feeling of wholeness: *“It gives a feeling of coming together…the soul gets back into the body again”.* They could feel where their body started and ended. For one patient, the invitation letter and the caring touch treatments were expressed as life-saving: *“When I got this invitation letter…it was the turning point in my life …because I had lost everything after the accident…I felt I had no force in life…and then came this letter…this was the turning point…oh, yes God!”*

#### Enjoying compassionate care

The therapist was important to the patients, and was described as creating a sense of security, a midwife who was letting the body rest in a moment of no responsibility: *“To just meet the therapist more or less like a newborn and she was the midwife, the beloved, so secure”.* Patients felt closely connected to the therapist, and being embraced by her safe hands, this made it possible to hand over responsibility: *“I know I will relax during the treatment and I hand over my body to her…I know she will take care of it”.* The caring touch treatment provided an opportunity to rest, feel relaxed and in harmony without pain: *“Just this…her energy flows into…yes, transfers…when she touches you… you regain strength”*. After the caring touch treatments, different perceptions of the body were experienced: *“I remember the first time I received massage, I felt a tingling in my body, and I felt, Oh God! I am starting to feel joy! And it was so…it was so striking…God, I felt happy”.*

#### Moments of pain alleviation

To some patients, pain totally disappeared during the caring touch treatments and for some hours afterwards. Some of the patients were even able to decrease their intake of analgesics: *“You forgot the pain totally, just when she presses just a little, it is not like ordinary massage, it feels really comfortable in the area where you have your pain. Yes, the pain decreases, you forget the pain…at the outset I took painkillers and was to some extent relaxed from the beginning. But then I tried the massage with fewer painkillers, and I felt that I could relax without medicine”.*

### VAS pain ratings and questionnaires

There were 27 patients that had complete VAS pain ratings at follow-up after 6 months. The results indicate both clinically and statistically significant improvements in VAS pain scores over time (Table 2). Exploratory outcomes of IES-R and EQ-5D index also showed significantly improved scores over time, whereas there were no statistically significant differences of SOC or EQ-5D VAS for self-rated health (Table 3).

**Table 2:** Pain ratings at baseline and follow-up after 6 months

	Baseline	Follow-up	
	Median (p25, p75)	Median (p25, p75)	*P*
VAS (*n* = 27)	60 (26, 74)	12 (6, 34)	<0.001

p25 and p75 denotes the 25th and 75th percentiles

**Table 3:** Questionnaire outcomes

	Baseline	Follow-up	
	Median (p25, p75)	Median (p25, p75)	*P*
IES-R (*n* = 23)	19 (8, 47)	9 (3, 21)	0.002
SOC (*n* = 26)	58 (53, 62)	59 (55, 62)	0.121
EQ-5D index (*n* = 26)	0.73 (0.66, 0.80)	0.80 (0.73, 0.80)	<0.001
EQ-5D VAS (*n* = 26)	73 (56, 80)	77 (70, 90)	0.092

p25 and p75 denotes the 25th and 75th percentiles

**Fig. 2:**
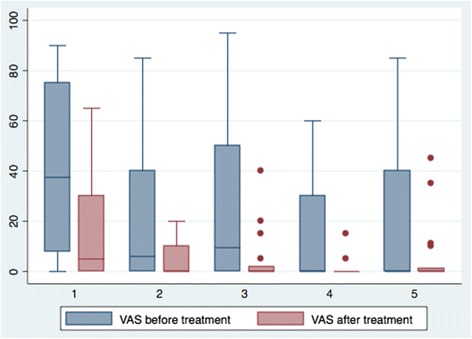
Box plot of visual analogue scale pain ratings (ranging from 0 no pain, to 100 worst pain) before and after five caring touch treatment sessions (medians with 25^th^ and 75^th^ percentiles and upper and lower adjacent values).

The patients additionally rated their pain on VAS before and after each treatment session with tactile massage or healing touch. The results indicate a decreased pain during the caring touch treatment. Approximately half (*n* = 18/41; 44 %) of the patients that were originally included received at least 5 (range 1 to 8) treatments with caring touch. This corresponds to a majority (*n* = 18/27; 65 %) of patients completing the study at follow-up after 6 months. A chart describing the before and after treatment pain ratings for these patients is depicted in Fig. 2.

## Discussion

### Findings

This mixed methods study captured patients’ lived experience after a MVA with minor or no physical injury and evaluated the subjective importance of an early intervention of caring touch. After a MVA - even if no or only minor physical injury is involved - the patient report existential needs which can be addressed with caring touch with the potential of creating a “bodily anchor” when recovering from a trauma. The following themes where identified; *a way of suffering*, *caring touch as a bodily anchor, enjoying compassionate care and moments of pain alleviation.*

#### The bodily anchor

Merleau-Pontys [24] ideas of the lived body illuminate and describe the taken-for-granted perception of being both body and soul in most moments. The subjective body is just “there” for us. We can never free ourselves from this embodiment, since the body connects us to or anchors us in the world [25]. Still, this anchorage may become lost during trauma, when the body is consciously or unconsciously disengaged, or as in the current study “switched off”, due to a natural reflex to protect the individual against trauma [26]. In a lifeworld perspective, we are our body and we experience ourselves, the world, and others through our lived, subjective body [24]. In the current study patients experienced that their body becomes an object in the world, something alien, unfamiliar to the patients, as bruises, pain and stiffness are seen as invading the body. Being in a MVA may be a life-threatening, traumatizing experience and the *meaning* of the experience may be as important as the physical injury itself [27]. For Merleau-Ponty, we get access to other human beings and things in the world through the communicative and interactive encounter, meaning that the world is something social [25]. In the caring encounter with caring touch in the current study, patients’ needs could be met, they were afforded an awareness of their bodies’ boundaries and got a sense of security.

#### A way of suffering

To be human is to suffer as a natural part of life, as too is the alleviation of suffering. The power to alleviate suffering is natural in oneself or together with others [28].

Suffering from sleep disturbance due to pain makes the body and mind tired, and loss of concentration weakens the body. This weakness and energy loss was also found in a study of Andersson et al. [29] where the patients still reported emotional suffering up to 2 years after the MVA. Some of the patients in the current study felt abandoned and of no importance after the trauma examination at the emergency department. This is line with a study of Doohan and Saveman [30] where passengers in a bus crash with minor injuries felt positive towards initial care, whereas later on their stay in hospital generated feelings of loneliness and emptiness. All this together indicates a need for the nursing staff to be aware of each patient’ s needs, especially when the patient has sustained a trauma where existential questions need to be met. When the existential needs and suffering of patients is not taken seriously, suffering may increase [31]. Findings in a study by Andersson et al. [29] indicate that lack of psychological support and information result in a higher rate of complications for patients sustaining an MVA with minor injury.

#### Enjoying compassionate care

The findings could be seen as a sign of the connectedness between body and soul. This intertwined body and soul is experienced as whole entity in contrast to being divided in two parts, “the lived-body”. In this intertwining, Merleau-Ponty characterizes a human being as a body-soul unit, continuously interacting with the world, in a lived relation to the world, and it is through this relationship that human experience is perceived [24, 25]. Touch is of great importance to human beings, nurturing the individual and restoring health and could even be experienced as life-saving [32]. The concept of touch in caring provides a profound ethical and existential aspect in the caring relationship between the nurse and patient and may contribute to the patients’ health in a positive manner. Natural and basic caring of the patient’s body in Eriksson’s nursing theory is called tending [33, 34]. Tending is one of the most profound elements of caring and entails taking care of the patient in body, soul and spirit. Caring touch makes is possible to become aware of the body, whereas normally the body is just “there” without deeper consideration. The caring touch treatments in current study seemed to influence self-awareness of the body and soul, and creating a feeling of being secure. This strengthening of the body-soul connection as a positive experience was also an outcome in a previous study of Lindgren et al. [35]. Present findings elucidate a caring encounter including connectedness between two fellow human beings were a “common meaning” on a deeper human level [36] or a “tactile room” [37] was opened. This tending care resulted in an alleviation of suffering and promotion of health during recovery from MVA with minor or no physical injury.

#### Moments of pain alleviation

The quantitative results suggest that caring touch may have a direct clinical impact on MVA patients’ VAS pain ratings, which were significantly decreased after treatment sessions with caring touch, and at follow-up, 6 months after the accident compared to baseline. In support of this, patients also expressed that they experienced pain alleviation during the caring touch treatments. Our findings are supported by previous studies reporting lower pain scores after tactile massage and healing touch [38–41].

### Integration of findings

Pain is a multi-dimensional experience including physical, psychological, social and existential pain, where the latter may cause severe suffering [42, 43]. When combining both qualitative and quantitative findings it seems that patients experienced less pain during caring touch. Interestingly, the experience of pain alleviation as well as reduced pain medication has been shown in previous studies of tactile massage and healing touch [13, 44].

Significant reduction of post traumatic stress over time in the scores of IES-R despite that patients’ reported experiences of intrusion and avoidance in their narratives 3 month after the accident. For the EQ-5D index, there were significant positive changes over time over time that indicate recovery; still, some patients’ narrated increased difficulties in mobility and anxiety. However, no differences were found in SOC and EQ-5D VAS for self-rated health. The reasons for this discrepancy are obscure and needs to be addressed by future studies. We also show that a trauma patient may benefit from being “anchored” in her/his body in order to feel secure. This is in line with the European Network for Traumatic Stress (TENTS) concluding that supporting people after a trauma should include strengthening their sense of security, strengthening their confidence, strengthening fellowship, creating tranquility and increasing their sense of hope [11]. When discussion the positive outcomes of this study one need to be aware that previous studies have shown that most patients exhibit symptoms shortly following trauma exposure and that the vast majority of patients will recover naturally [45]. Previous research indicates that crisis interventions following traffic accidents display varying results from no, or uncertain outcomes to positive effects [46, 47]. The influence of the touch therapist need to be addressed as the patient-provider connectedness is believed to have a positive influence on patient health outcomes [48]. However, in previous randomised controlled trials with touch verses various interventions the results in the touch cohorts shows less anxiety and pain [38, 39, 41, 49].

### Methodological considerations

The patients in the current study were conveniently invited to participate. This resulted in an unbalanced group in terms of gender which limits the generalizability of the findings. In addition, the lack of a control group makes it difficult to generalize about the specific effect of the intervention itself. However, the trend of predominant female participation has also been seen in a previous study of MVA [4]. Women are also more likely to use complementary treatments [44, 50, 51]. Hence, it is recommended that future studies prioritize resources to enable proper handling of clinical logistics and other constraints surrounding the procedures of recruiting patients, so as to be able to conduct a standard randomized controlled trial, and where a stratified randomization procedure is performed to balance for gender. Preferably, future investigations should keep the mixed methods approach to analysis [14]. This was a useful method to verify and describe the clinical importance of our findings and the patients’ experiences and attitudes in relation to e.g., the improved VAS pain ratings that emerged in the current study.

The VAS pain outcome measure was highly applicable in terms of its long-term established use in the clinical emergency care setting. The IES-R is a common instrument in trauma research as well as SOC and both are preferable to use in further studies of motor vehicle accident patients [52]. When interviewing patients, a conscious effort was made to temporarily suspend my pre-understanding of both tactile massage and healing touch to establish trustworthiness, i.e. meaning that we did not take any answer for granted. This also requires openness towards the lived experience of the patients. Both the first (FA) and second (MA) author reined in our pre-understanding during the text analysis, as we did not share the same pre-understanding. The credibility of the results in the current study may have been affected by the fact that the patients who joined the study in order to receive caring touch probably already were positive toward touch.

## Conclusions

Sustaining a MVA with minor or no physical injury can imply emotional and physical reactions. Therefore it is not surprising that a caring touch intervention during first weeks following the accident was associated with positive result, both in terms of improved pain ratings (VAS) and in lived experiences by interviews. Caring touch seems to responding to the need of the individual, who, when experiencing dissociation, may regard caring touch as a physical anchor, holding the self together and creating bodily boundaries. However, even if the results of caring touch may have positive outcomes there is a need to be aware of the potential impact of the therapist in the patient-provider relationship.

## Declarations

### Ethics approval and consent to participate

The research project was approved by the Regional Research Ethics Committee in Stockholm, Sweden (Dnr 2010/514-31/2). Ethical principles have been performed in accordance to the Declaration of Helsinki, usefulness in relation to risks, integrity, non-malfeasance and respect for human dignity were considered. Patients were assured that they could withdraw from the study at any time and that it would not affect their care. Patients received oral and written information about the study and were informed about the nature and purpose of the study and the intended use of data.

### Consent for publication

Written informed consent to participate and for results to be publish at group level was obtained from all participants.

### Availability of data and materials

The data were coded and only the research group was permitted to have access to the data.
